# Limonoid compounds from *Xylocarpus granatum* and their anticancer activity against esophageal cancer cells

**DOI:** 10.1111/1759-7714.13455

**Published:** 2020-05-25

**Authors:** Li Jing, Li Feng, Zhiguo Zhou, Shuai Shi, Ruoying Deng, Zhicong Wang, Yibing Liu

**Affiliations:** ^1^ Department of Medical Oncology Fourth Hospital of Hebei Medical University Shijiazhuang Hebei Province China; ^2^ Hebei Medical University Hebei Medical University Shijiazhuang Hebei Province China

**Keywords:** Apoptosis, esophagus cancer, Limonoid compound, proliferation, xylogranatin C

## Abstract

**Background:**

To investigate the anticancer effects of limonoid compounds that were isolated and purified from *Xylocarpus granatum* fruits on human esophageal cancer (EC) cells. A structure‐activity relationship experiment was designed to identify the functional moiety of limonoid compounds identified as being critical for its anticancer activity.

**Methods:**

Eca109 cells were cultured in RPMI1640 medium and treated with limonoid compounds. Cell proliferation was determined by the MTT assay in vitro. Eca109 cells apoptosis was analyzed by by flow cytometry after being treated with xylogranatin C. The expression of p53, Bax, bcl‐2, caspase‐3 and GRP78 in Eca109 cells after xylogranatin C treatment was examined by western blot assay.

**Results:**

Four linonoid compounds strongly inhibited the cellular proliferation of Eca109 cells. Xylogranatin C was the strongest inhibitor, whose inhibitory effect was comparable to that of the well‐known chemotherapeutic agent, cisplatin. Furthermore, xylogranatin C might induce Eca109 cell apoptosis through joint effects on multiple pathways, including the death receptor and endoplasmic reticulum pathways. Additionally, xylogranatin C suppressed tumor cell proliferation by upregulating miR‐203a expression in Eca109 cells.

**Conclusions:**

Xylogranatin C induced Eca109 cellular apoptosis and exerted antitumor activity. Xylogranatin C suppressed tumor cell proliferation by upregulating miR‐203a expression in Eca109 cells.

## Introduction

Esophageal cancer (EC) is one of the leading causes of cancer‐related death worldwide. Recently, advances have been made in the diagnosis and treatment of EC which have improved the prognosis of patients with early stage EC. However, EC is usually diagnosed at an advanced stage and its prognosis remains dismal. Moreover, EC is highly resistant to radiotherapy/chemotherapy, which is the main reason for its poor prognosis. Thus, experts and scholars worldwide should make great efforts to more deeply understand the pathogenesis and molecular mechanism of EC, and to identify natural medicinal agents with definitive curative effects without undesirable side effects.

Medicinal herbs are rich sources of potential anticancer lead compounds.^1^ Further structural modifications of these lead compounds allow the development of better chemotherapeutic agents with higher potency and improved safety profiles^.^ ^2^ *Xylocarpus granatum* is a plant of neem, widely distributed in the Indian Ocean and along the Southeast Asia coast, and widely distributed along the coast of Hainan province in China. The published literature has reported that its extracts display antitumor activity^.^ ^3^, ^4^ *X. granatum* is rich in lemon bitter compounds, and the compound has a variety of biological activities, including bacteriostatic, insecticidal, tumoricidal, and antiviral activities, among others.^5^, ^6^, ^7^ Since Taylor reported the first lemon bitter compound Godunin in 1965, research on lemon bitter compounds has attracted widespread attention.^8^ In this study, we isolated nine compounds from the root of *X. granatum*, including odoratone, hispidol B, spicatin, 7‐deacetyl‐7‐oxogedunin, xylogranatin C, xylogrnantin C, xylocarponoid A, proceranolide, and xylogranatin A. We investigated their effects on cell proliferation and apoptosis of EC cells.

### Methods

**Figure 1:**
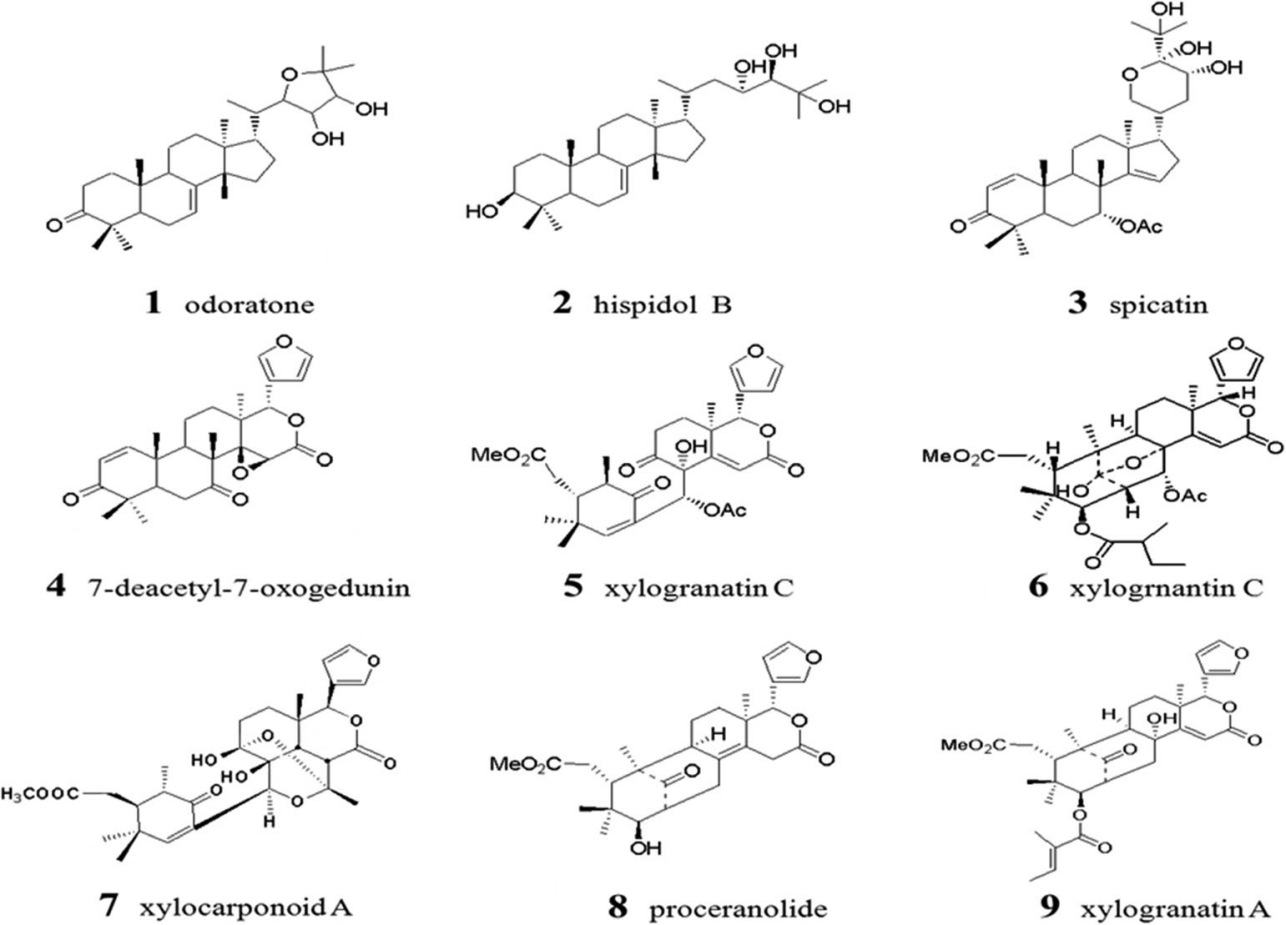
Chemical structures of nine limonoid compounds from *Xylocarpus granatum*.

Nine species of lemon bitter compounds including odoratone (1), hispidol B (2), spicatin (3), 7‐deacetyl‐7‐oxogedunin (4), xylogranatin C (5), xylogrnantin C (6), xylocarponoid A (7), proceranolide (8), and xylogranatin A (9) were extracted and purified from *X. granatum* by the Department of Natural Pharmaceutical Chemistry of Hebei Medical University, China. The chemical structure was determined by NMR spectroscopy and mass spectrometry and all compounds had a purity of more than 99%. The structure of these compounds is shown in Fig 1.

### Cell culture

Eca109 cells were obtained from the Fourth Hospital of Hebei Medical University, China. Cells were cultured in RPMI1640 medium (Invitrogen, Carlsbad, CA, USA) and this was supplemented with 10% heat‐inactivated FBS (Invitrogen, Carlsbad, CA, USA), 100 U/mL penicillin, and 100 ug/mL streptomycin, and subcultured upon reaching confluence.

### Cell proliferation assay

Cell proliferation was determined by MTT assay. Logarithmically proliferating cells were collected after tripsinization and plated at a density of 1.0 × 10^4^ cells per well in 96‐well plates with 100 μL of the medium containing a compound at the indicated concentrations. Two days after treatment, cells were incubated with 100 μL of the medium containing 0.5 mg/mL MTT for four hours. Then, 100 μL of stop solution (2‐propanol containing 80 mM HCl) was added to each well and the plates were shaken for 10 minutes to dissolve the resultant formazan crystals. Finally, the absorbance was measured at a wavelength of 570 nm with a reference wavelength set at 655 nm. The survival rate was calculated according to the following formula: Growth survival rate (%) = [A570(experimental)/A570(control)] × 100%.

### Apoptosis analysis by flow cytometry assay

Eca109 cells were treated with 10 and 20 μmol/L xylogranatin C for 12 and 24 hours. Then, 1.0 × 10^7^ cells were harvested, washed twice with PBS, and resuspended in 300 μL of binding buffer. Following the manufacturer's instructions, Annexin V and PI were added to each sample and incubated for 30 minutes. Finally, each sample was evaluated for apoptosis in binding buffer by using a FACS flow cytometer (Becton‐Dickinson).

### Expression of p53, Bax, bcl‐2, caspase‐3 and GRP78 by western blot assay

Eca109 cells were plated at a density of 1.0 × 10^7^ cells per well with separate groups of RPMI‐1640, DMSO and xylogranatin C at the indicated concentrations. Cell scraper scratches across each group of cells were conducted, and the supernatant was the total cell protein after RIPA buffer was added to each group of cells. The extracted proteins from each group were added to 1 × loading buffer. The protein gel after electrophoresis was completely transferred to the PVDF membrane by the Bio‐rad device. Monoclonal antibodies and β‐actin was added in TTBS. Development was completed using the Odyssey two‐color infrared imaging system by placing the PVDF film in the film sweep instrument, following which, the results of the scan were loaded into the computer, and analyzed by Image‐j software, which was used to process the images. The β‐actin protein loading control value and experimental group numerical ratio were used to determine the protein expression levels.

### Flip effect of cysteine enzyme inhibitor on inhibiting Eca109 cell proliferation by xylogranatin C

Eca109 cell proliferation was determined by the MTT assay. Cells were collected after tripsinization and plated at 1.0 × 10^4^ cells per well in 96‐well plates with a cysteine enzyme inhibitor, cisplatin and xylogranatin C. Two days after treatment, cells were incubated with MTT for four hours and the MTT assay procedure was followed as described above.

### qRT‐PCR and assay of miR‐203a expression in xylogranatin C treated Eca109 cells

Total RNA was isolated from Eca109 using TRIzol reagent and cDNA were synthesized using the TaqMan Micro‐RNA Reverse Transcription Kit. The primers and reaction conditions are demonstrated in Table 1. The expression levels of miRNAs were normalized with U6 by the 2 –^ΔCT^ method.

**Table 1:** The antiproliferative activity of four limonoid compounds on Eca109 cells

	IC50 (μmol/L)	
Compounds	Eca109	
Cisplatin	12.98	
Compound 3	37.49	
Compound 4	29.02	
Compound 5	9.50	
Compound 7	13.34	

The half‐maximal inhibitory concentrations (IC50, μmol/L) of compound 3, 4, 5 and 7 toward the indicated cell‐lines were calculated based on the results of an MTT assay (*n* = 3).

### Statistical analysis

Statistical analysis was performed with Origin 7.0 software for the cell proliferation assay. The results of the inhibitory rate are expressed as mean ± S.D. The experimental compounds were fitted with a Hill mathematical model for the concentration‐effect curve of tumor cells to calculate the IC50 values of the drug of interest. Statistical analysis was performed with SPSS version 19.0 software package (SPSS Company, Chicago, Illinois, USA) for flow cytometry, the expression of related genes and western blot assays. Data are expressed as mean ± standard deviation to compare single factor variance analysis (ANOVA) of each group, and the minimum significant difference method (least significant difference, LSD) was compared. An alpha value of *P* < 0.05 was considered to be statistically significant for all tests.

## Results

### Inhibitory effects of nine tested compounds on proliferation of Eca109 cells

**Figure 2:**
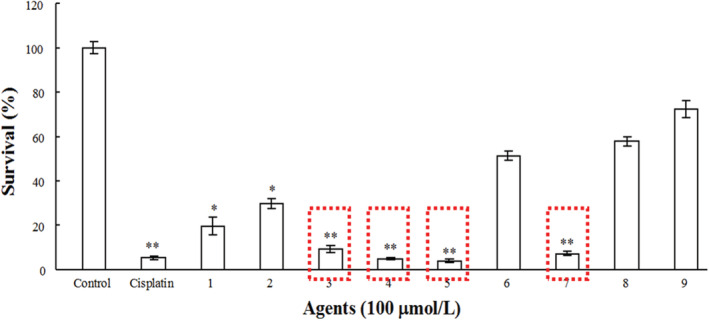
The effects of nine limonoid compounds from *Xylocarpus granatum* and the proliferation of Eca109 cells (χ ± s, *n* = 3). *P < 0.05, **P < 0.01 as compared with the control group.

Eca109 cells that were cultured in vitro were tested for their inhibition by nine compounds and cisplatin. Spicatin (3), 7‐deacetyl‐7‐oxogedunin (4), xylogranatin C (5) and xylocarponoid A (7) inhibited cellular proliferation, and cell survival rates were 9.19, 5.01, 4.04% and 7.15%, respectively. Other compounds that included odoratone (1), hispidol B (2), proceranolide (8) and xylogranatin A (9) showed lower or no inhibitory activity on the proliferation of Eca109 cells (Fig 2). By the logarithmic regression equation of tumor cell proliferation and survival rate to the concentration of compound three, four, five and seven, the IC50 of these four compounds on Eca109 cells was determined (Table 2). Combined with the above results, the inhibitory effect of xylogranatin C on cell proliferation/survival was higher than that of the chemotherapeutic drug cisplatin.

**Table 2:** Primer sequences and reaction conditions qRT‐PCR for miR‐203a

Type	Primer sequence	Annealing Temperature (tl)	Product size (bp)
qRT‐PCR	RT: 5′‐GTCGTATCCAGTGCAGGGTCCGAGGTATT		
	CGCACTGGATACGACCTAGTGGT‐3′		
miR‐203a	F: 5′‐GTGCAGGGTCCGAGGTATT‐3′		
	R:5′‐GCCGCGTGAAATGTTTAGGACCAC‐3′	55	
	RT:5′‐AACGCTTCACGAATTTGCGT‐3′		
U6	F: 5′‐CTCGCTTCGGCAGCACA −3′		
	R:5′‐AACGCTTCACGAATTTGCGT‐3′	55	
miR‐203a	MR: 5′‐AAACGACTAAACTCCGAACG‐3′	58	166
	UF:5′‐GGGTTGTGGATTAGTT‐3′		
	UR‐5′‐AAACAACTAAACTCCAAACA‐3′	52	166

F, forward primer; MR, methylated reverse primer; R, reverse primer; RT, reverse transcription primer; UF, unmethylated forward primer; UR, unmethylated reverse primer.

### Induction of cellular apoptosis by xylogranatin C

**Figure 3:**
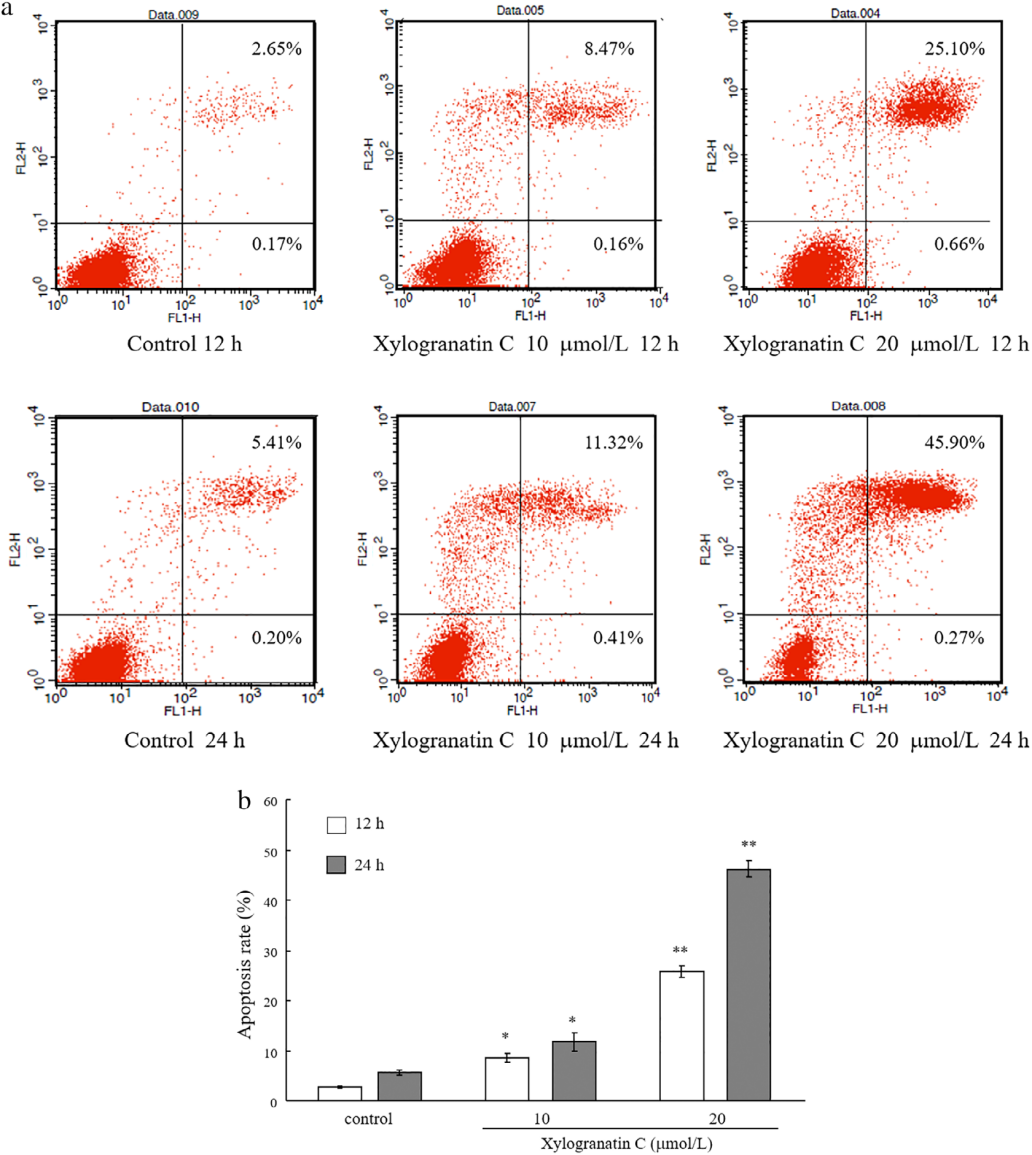
(**a**) Detection of apoptosis in Eca109 cells by flow cytometry. Values are expressed as mean ± standard deviation (SD) of three independent experiments. *P < 0.05; **P < 0.01 (**b**) as compared with the control group. (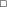) 12 hours, (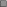) 24 hours.

**Figure 4:**
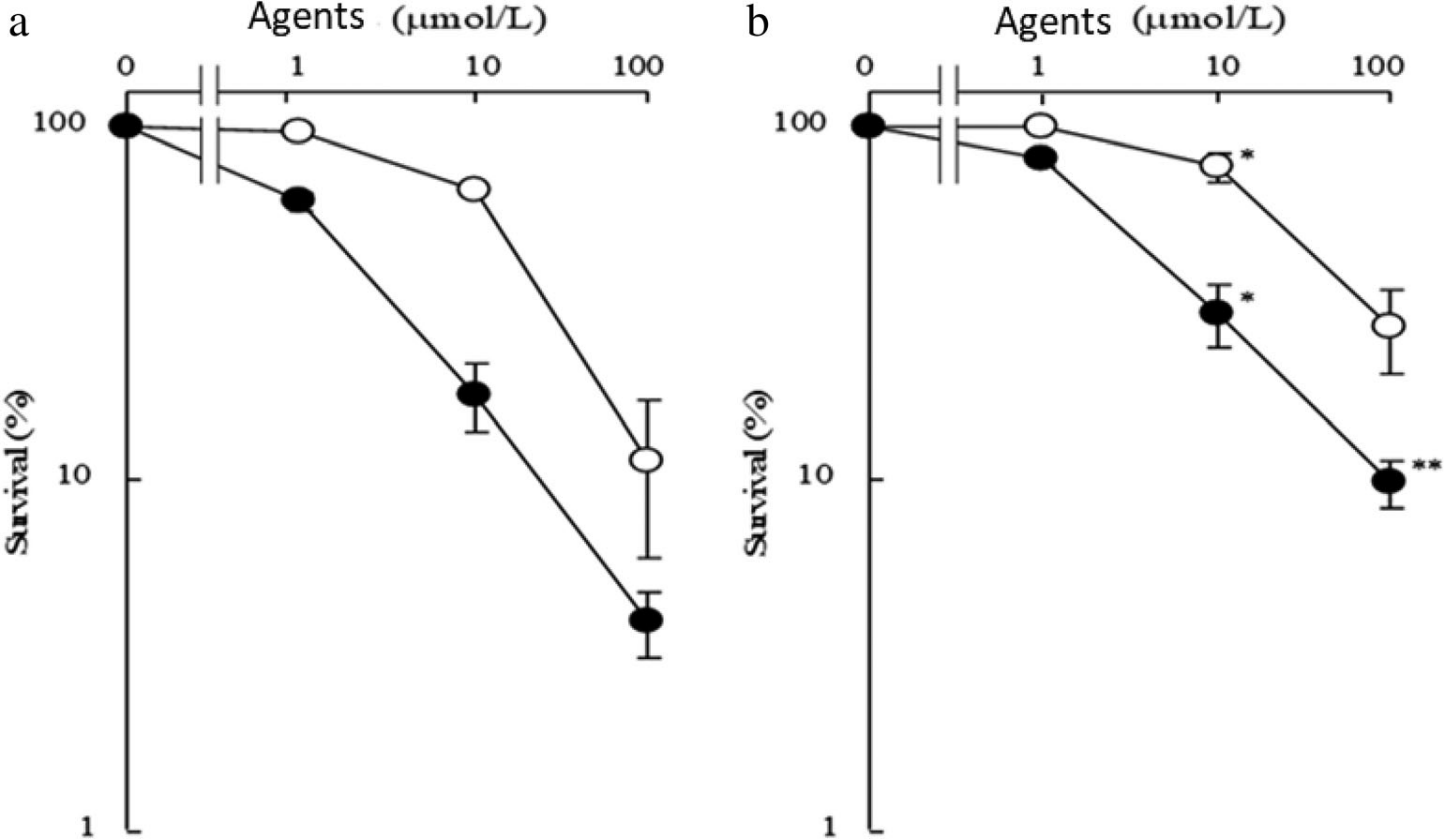
Effect of the cysteamine inhibitor on xylogranatin C inhibition of the proliferation of Eca109 cells. (**a**) inhibitor (−); (**b**) inhibitor (+). Data are expressed as mean ± SD (*n* = 3). **: *P* < 0.01 and *: *P* < 0.05 as compared with the inhibitor (−) group. (**a**) (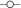) Cisplatin, (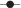) Xylogranatin C; (**b**) (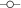) Cisplatin, (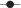) Xylogranatin C.

To determine whether the anticancer activity of xylogranatin C was associated with apoptosis, Eca109 cells were analyzed by flow cytometry assay. As shown in Fig 3, the number of apoptotic cells was increased by xylogranatin C (at 10, or 20 μmol/L) after 12 and 24 hours of treatment. Further, a general caspase inhibitor was examined for its protection of cell death induced by xylogranatin C. Treating Eca109 cells with xylogranatin C in the presence of a caspase inhibitor, restored cell survival, which suggested that induction of caspase‐dependent apoptosis was involved in the antiproliferative activity of xylogranatin C (Fig. 4).

### Mechanism guiding apoptosis by xylogranatin C

**Figure 5:**
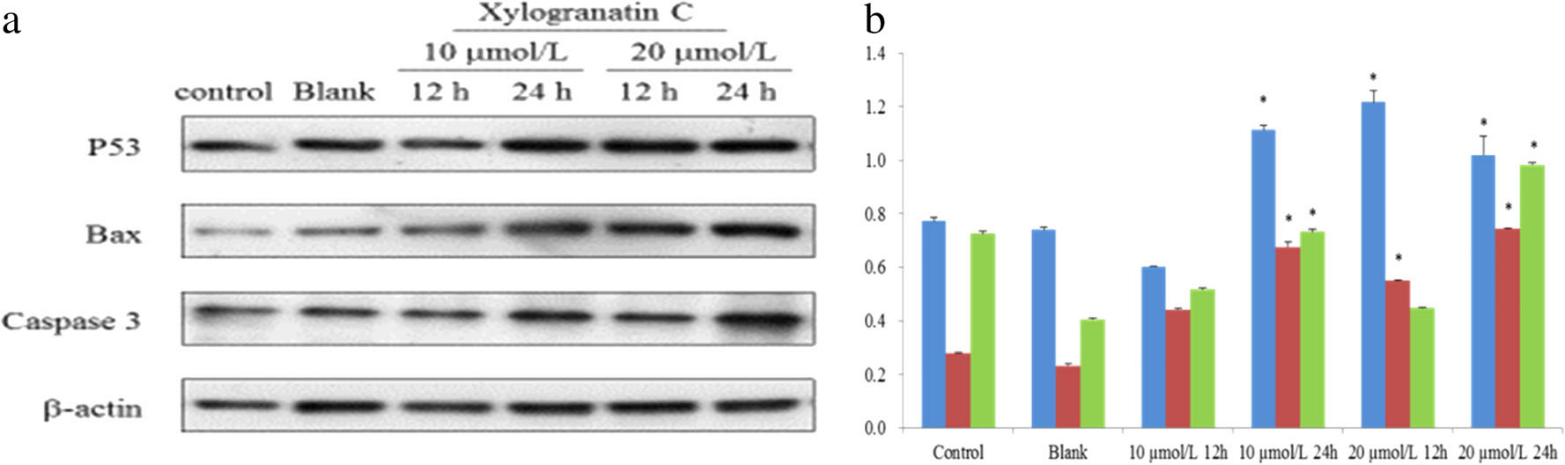
(**a**) Effects of xylogranatin C on the expression of apoptosis‐associated proteins in Eca109 cells detected by WB. β‐actin was used as an internal control. Data are expressed as mean ± SD (*n* = 3). *: *P* < 0.05; **: *P* < 0.01, as compared with the control group (**b**). (**b**) (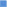) P53, (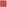) BAX; (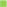) Caspase‐3.

**Figure 6:**
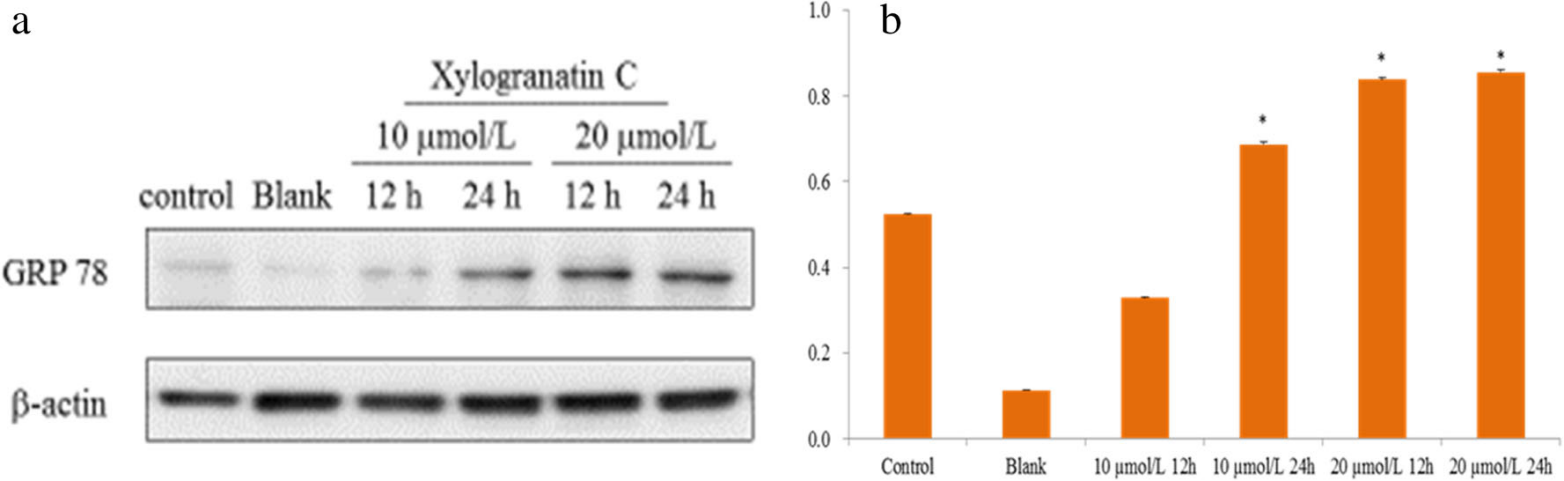
(**a**) Effects of Xylogranatin C on GRP 78 expression in Eca109 cells following ER stress. β‐Actin was used as an internal control. Data are expressed as mean ± SD (*n* = 3). *: *P* < 0.05, as compared with the control group (**b**). (**b**) (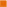) GRP78.

To further investigate the apoptosis induction by xylogranatin C was critical, and we subjected Eca109 cells to western blot analysis to determine the mechanism of guiding apoptosis by Xylogranatin C. After Xylogranatin C treatment at a final concentration of 10 or 20 μmol/L for 24 hours, the expression of p53, Bax and caspase‐3 were found to be markedly increased as compared with the control group (*P* < 0.05; Fig 5). After xylogranatin C treatment at a final concentration of 10 or 20 μmol/L for 12 and 24 hours, the expression of GRP78 in the 10 umol/L and 20 μmol/L treated groups for 12 and 24 hours was found to be markedly increased as compared with the control group (*P* < 0.05; Fig 6).

### Relevance of functional expression of miR‐203a following xylogranatin C treatment of Eca109 cells

**Figure 7:**
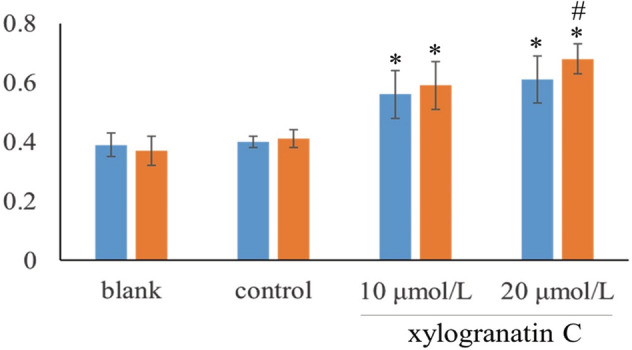
Xylogranatin C expression on miR‐203a in Eca109 cells after 12 or 24 hours (*P < 0.01), as compared with the blank and control groups. #*P* < 0.05, as compared with the 12 hours group. (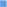) 12 hours, (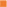) 24 hours.

miR‐203a expression levels were upregulated in Eca109 cells after 10 μmol/L and 20 μmol/L xylogranatin C treatment for 12 and 24 hours as compared with the control group (*P* < 0.01). In addition, with increasing concentration and extension in time, the expression was markedly increased, and the difference in expression seen between the 12 and 24 hour groups were significant (*P* < 0.05; Fig 7).

## Discussion

*X. granatum* is a plant of the genus Neem. Lemon bitter compounds is an important type of chemical compound found in *X. granatum*. Lemon bitter is a highly oxidized four‐drop triterpenoid, and some reports from the domestic literature have shown that it has many biological activities, such as having an antitumor effect. In our present work, compound 3, 4, 5 and 7 was targeted against Eca109 cells, showing inhibition of cellular proliferation activity. However, the inhibitory effect of xylogranatin C on cellular activity was higher than that of the well‐known chemotherapy drug cisplatin.

Numerous studies have reported that many bioactive components in traditional herbal preparations have obvious antitumor efficacy, although the mechanism of action is complex. Also numerous components achieve their biological activity by inducing the apoptosis of tumor cells. Feng *et al*.^9^ conducted a study where they isolated and purified compound C22 from the Sea Moth, and this compound displayed activity against human cervical cancer Hela cells. Compound C22 displayed a strong inhibitory effect against the proliferation of Hela cells, in a mechanism that might have been mediated via the Bcl‐2/caspase pathway to induce Hela cell apoptosis. Hongli *et al*.^10^ investigated the R50 compound of *Polygonum chinensis*, and found that R50 decreased the survival rate of human colorectal cancer HCT16 and HT29 cells, and did so in a mechanism that induced human colorectal cancer HCT16 and HT29 cell apoptosis via the glutathione dependent signaling pathway. Several studies have also shown that the extraction and purification of monomer compounds from other Chinese herbal medicines, such as artesunate^,^ ^11^ wogonin^,^ ^12^ paclitaxel compounds^13^ and others, might also be effective in driving an antitumor activity by causing apoptosis of tumor cells.

The results of flow cytometry demonstrated that xylogranatin C at 10 and 20 μmol/L displayed dose‐and time‐dependent effects in treated Eca109 cells as compared with the blank control group. We speculated that the inhibitory effect of xylogranatin C on the proliferation of Eca109 EC cells might be achieved by inducing apoptosis. The cysteine enzyme family (caspase family) plays an irreplaceable role in the initiation and development of apoptosis. Caspase‐3, −6 and −7 are all involved in apoptosis, of which Caspase‐3 is known as the ultimate executor of apoptosis, and plays an important role in the exogenous and endogenous apoptosis pathways^.^ ^14^ Caspase family inhibitors can increase the inhibitory effect of xylogranatin C on the proliferation of Eca109 cells in human EC, and such studies verify that xylogranatin C disrupts the proliferation of antitumor cells by inducing tumor cell apoptosis.

There are two main pathways that lead to apoptosis and are broadly referred to as the endogenous and exogenous pathways. P53 protein is one of the key factors of the endogenous pathway of apoptosis. It is a cellular receptor that receives various external stimuli to the cell. Due to the presence of the P53 binding loci in the promoter of the Bax gene, P53 can induce apoptosis by directly raising the expression levels of the Bax protein in the cell, or by inhibiting the protein expression of Bcl‐2 and promoting the expression of Bax and Bcl‐xl.^15^ Increased expression of intracellular Bax protein directly leads to increased permeability of mitochondrial outer membranes, which is also a vital factor in inducing apoptosis.

GRP78 is mainly found in the endoplasmic reticulum as a molecular companion of the endoplasmic reticulum^.^ ^16^ GRP78 belongs to a member of the heat shock protein family and plays an important role in the activation of proto‐oncogenes. Recent studies have shown that endoplasmic reticulum stress pathways induce apoptosis. In this current study, western immunoblotting was used to observe the upward effect of the lemon bitter compound xylogranatin C on the expression of four protein factors that included: P53, Bax, Caspase‐3 and GRP78 in Eca109 cells. We found that after xylogranatin C treatment of Eca109 cells for 12 and 24 hours, the expression of P53, Bax, Caspase‐3 and GRP78 protein all increased, and the expression levels of the GRP78 protein increased gradually, showing both time and dose dependence. It is speculated that xylogranatin C might induce tumor cell apoptosis via the endogenous apoptosis pathway and the endoplasmic reticulum pathway.

Research on the genes of lemon bitter compounds has not been previously reported.^19^, ^20^ MiR‐203a was dysregulated in multiple malignant carcinomas such as laryngeal squamous cell carcinoma, hepatocellular carcinoma, oral cancer cells, cervical cancer, etc.^21^, ^22^, ^23^, ^24^, ^25^, ^26^, ^27^, ^28^, ^29^, ^30^, ^31^, ^32^, ^33^, ^34^, ^35^, ^36^ However, up or downexpression of miR‐203a in different carcinomas has been reported indicating that it may play a different biological function in different cancer cells.^37^, ^38^, ^39^, ^40^, ^41^, ^42^, ^43^

More and more evidence had demonstrated that miRNA may play an important role in the progression and metastasis of human cancer.^17^, ^18^ MicroRNA (miRNAs) in different malignant tumors has been considered as a putative therapeutic and prognostic molecular marker. Studies have shown that^44^ the expression of miR‐203 in 125 non‐small cell lung cancer tissues is lower than that of adjacent tissues, and that the survival period of patients with low expression is shorter. Wan *et al*.^45^ found that in primary hepatocellular carcinoma, the expression levels of miR‐203 was associated with the total survival period. In addition, the prognosis was poor in patients with low expression of miRNA‐203. Previous studies have found that esophageal squamous cell carcinoma (ESCC) patients with low expression of miR‐203a have a poor five‐year survival rate. The recovery of miR‐203a expression using drugs or other methods might represent a novel direction in the treatment of ESCC metastasis. Therefore, the expression of miRNA‐203 in Eca109 human esophageal carcinoma cells was found following treatment with xylogranatin C. It was found that the expression of the miR‐203a gene increased gradually with increases in both time and concentration. Thus, xylogranatin C‐induced expression of miR‐203a in Eca109 EC cells could be increased by restoring the expression of the miR‐203a gene. Previous studies have shown that increased expression of miR‐203a could inhibit the proliferation and invasiveness of ESCC.^46^ Thus, xylogranatin C blocks the proliferation of malignant cancer cells, which might be achieved by raising the relative expression levels of the miR‐203a gene.

Through these studies, we can speculate that the inhibitory effect of xylogranatin C on Eca109 cells is due in large part to its role as an antitumor agent that disrupts the cellular proliferation of cancer cells and does so via the endogenous apoptosis pathway. The involved mechanism is thought to operate by increasing the expression of the P53 protein, and then further increase Bax apoptosis‐inducing protein levels, which results in the downstream activation and increased expression of Caspase‐3, and eventually promotes apoptosis of tumor cells. The role of the endoplasmic reticulum stress pathway is also one of the mechanisms involved in the apoptosis of Eca109 cells following treatment with lemon bitter compounds. However, apoptosis is a multifactorial process and requires the participation of multiple pathways. Thus, whether there are also other ways to induce tumor cell apoptosis by xylogranatin C still needs further exploration. Xylogranatin C, at different concentrations, can increase the expression of the miR‐203a gene in EC Eca109 cells, which might also represent an additional mechanism by which xylogranatin C induces antitumor proliferation.

In conclusion, Lemon Spicatin (3), 7‐deacetyl‐7‐oxogedunin (4), Xylogranatin C (5) and Xylocarponoid A (7) show remarkable inhibitory effects on the proliferation of EC cells. The inhibitory effect of xylogranatin C on cellular behavior was higher than that of the chemotherapeutic drug cisplatin. Moreover, xylogranatin C has significant apoptosis‐inducing activities in esophageal carcinoma cells. Xylogranatin C blocks the proliferation of cancer cells by raising the expression levels of miR‐203a in Eca109 EC cells.

## Disclosure

The authors declare that there no conflicts of interest.

## References

[1] Ho JW , Leung YK , Chan CP . Herbal medicine in the treatment of cancer. Curr Med Chem Anticancer Agents 2002; 2 (2): 209–14. 12678744 10.2174/1568011023354164

[2] Lee KH . Discovery and development of natural product‐derived chemotherapeutic agents based on a medicinal chemistry approach. J Nat Prod 2010; 73 (3): 500–16. 20187635 10.1021/np900821e PMC2893734

[3] Li M , Liu X , Guo SH *et al* Study on inhibition of proliferation activity of brain metastatic cells in human breast cancer by Neem lactone. Chin PLA Med J 2013; 34 (8): 865–8.

[4] Chang HP , Wang SM , Huo CH *et al* Study on inhibition of proliferation activity and mechanism of tumor cells in human lung cancer by Neem lactone compound. China Pharmacol Bull 2012; 28 (6): 807–10.

[5] Li J , Li MY , Bruhn T , Götz DC , Xiao Q , Satyanandamurty T , Wu J , Bringmann G . Andhraxylocarpins A‐E: Structurally intriguing limonoids from the true mangroves *Xylocarpus* granatum and *Xylocarpus* moluccensis. Chem Eur J 2012; 18 (45): 14342–51. 23008237 10.1002/chem.201202356

[6] Wang R , Ma TM , Liu F , Gao HQ . Research progress on pharmacological action and clinical application of Stephania TetrandraeRadix. China J. of Chinese Materia Medica 2017; 42 (4): 634–639. 10.19540/j.cnki.cjcmm.20170121.024 28959829

[7] Huo CH , Guo D , Shen LR *et al* A new lemon bitter compound in the seeds of Neem. Chin Herb Med 2010; 41 (2): 176–8.

[8] Zhang H , Wang X , Chen F , Androulakis XM , Wargovich MJ . Anticancer activity of limonoid from khaya senegalensis. Phytother Res 2007; 21 (8): 731–4. 17450502 10.1002/ptr.2148

[9] Li F , Pei SF , Shi XA *et al* Inhibitory mechanism of Hela cell proliferation and induction of human cervical cancer by active site of sea moth. China Pharmacol Bull 2017; 33 (11): 1546–52.

[10] Yang HL , Li RJ , Li ZM , Zhang RC , Li BS , Sun ZX . Mechanism of apoptosis of human colorectal cancer cells induced by R50 of Polygonum chinensis. Chin J Pharmacol Toxicol 2014; 28 (1): 51–6.

[11] Zhang Y , Wang C , Wang H , Wang K , Du Y , Zhang J . Combination of Tetrandrine with cisplatin enhances cytotoxicity through growth suppression and apoptosis in ovarian cancer in vitro and in vivo. Cancer Lett 2011; 304 (1): 21–32. 21333438 10.1016/j.canlet.2011.01.022

[12] Feng R , Zhai WL , Yang HY , Jin H , Zhang QX . Induction of ER stress protects esophagus cancer cells against apoptosis induced by cisplatin and doxorubicin through activation of p38 MAPK. Biochem Biophys Res Commun 2011; 406: 299–304. 21320468 10.1016/j.bbrc.2011.02.036

[13] Wang X , Gong W , Qing H *et al* p21‐activated kinase 5 inhibits camptothecin‐induced apoptosis in colorectal carcinoma cells. Tumour Biol 2010; 31 (6): 575–82. 20567954 10.1007/s13277-010-0071-3

[14] Liu C , Yang J , Fu W *et al* Coactvation of the PI3k/Akt and ERK signaling pathways in PCB153‐induced NF‐κB activation and caspase inhibition. Toxicol Appl Pharmacol 2014; 277 (3): 270–8. 24726520 10.1016/j.taap.2014.03.027

[15] Han HS , Park YM , Hwang TS . Differential expression of Bcl‐2,Bcl‐X,and p53 in colorectal cancer. J Gastroenterol Hepatol 2006; 21: 1108–14. 16824061 10.1111/j.1440-1746.2006.04325.x

[16] Fu R , Yang P , Wu HL , Li ZW , Li ZY . GRP78 secreted by colon cancer cells facilitates cell proliferation via PI3K/Akt signaling. Asian Pac J Cancer Prev 2014; 15 (17): 7245–9. 25227822 10.7314/apjcp.2014.15.17.7245

[17] Bertoll G , Cava C , Castiglioni I . The potential of miRNAs for diagnosis, treatment and monitoring of breast cancer. Scand J Clin Lab Invest 2016; 76 (Suppl. 245): S34–9. 10.1080/00365513.2016.1208444 27435502

[18] Oom AL , Humphries BA , Yang C . MicroRNAs: Novel players in cancer diagnosis and therapies. Biomed Res Int 2014; 2014: 959461. 25101302 10.1155/2014/959461 PMC4101974

[19] Mirnezami AH , Pickard K , Zhang L , Primrose JN , Packham G . MicroRNAs: Key players in carcinogenesis and novel therapeutic targets. Eur J Surg Oncol 2009; 35: 339–47. 18644693 10.1016/j.ejso.2008.06.006

[20] Xue J , Niu J , Wu J , Wu ZH . MicroRNAs in cancer therapeutic response: Friend and foe. World J Clin Oncol 2014; 5: 730–43. 25302173 10.5306/wjco.v5.i4.730 PMC4129536

[21] Tian L , Li M , Ge J *et al* MiR‐203 is downregulated in laryngeal squamous cell carcinoma and can suppress proliferation and induce apoptosis of tumours. Tumour Biol 2014; 35: 5953–63. 24682952 10.1007/s13277-014-1790-7

[22] Liu Y , Ren F , Rong M , Luo Y , Dang Y , Chen G . Association between underexpression of microrna‐203 and clinicopathological significance in hepatocellular carcinoma tissues. Cancer Cell Int 2015; 15: 62. 26109910 10.1186/s12935-015-0214-0 PMC4479344

[23] Lee SA , Kim JS , Park SY *et al* miR‐203 downregulates Yes‐1 and suppresses oncogenic activity in human oral cancer cells. J Biosci Bioeng 2015; 120 (4): 351–8. 25910964 10.1016/j.jbiosc.2015.02.002

[24] Xu M , Gu M , Zhang K , Zhou J , Wang Z , Da J . miR‐203 inhibition of renal cancer cell proliferation, migration and invasion by targeting of FGF2. Diagn Pathol 2015; 10: 24. 25890121 10.1186/s13000-015-0255-7 PMC4419389

[25] Liao H , Bai Y , Qiu S *et al* MiR‐203 downregulation is responsible for chemoresistance in human glioblastoma by promoting epithelial‐mesenchymal transition via SNAI2. Oncotarget 2015; 6: 8914–28. 25871397 10.18632/oncotarget.3563 PMC4496192

[26] Mao L , Zhang Y , Mo W , Yu Y , Lu H . BANF1 is downregulated by IRF1‐regulated microRNA‐203 in cervical cancer. PLoS One 2015; 10: e0117035. 25658920 10.1371/journal.pone.0117035 PMC4319761

[27] Qu Y , Li WC , Hellem MR *et al* MiR‐182 and miR‐203 induce mesenchymal to epithelial transition andself‐sufficiency of growth signals viar epressing SNAI2 inprostate cells. Int J Cancer 2013; 133: 544–55. 23354685 10.1002/ijc.28056

[28] Tang G , Wu J , Xiao G , Huo L . miR‐203 sensitizes glioma cells to temozolomide and inhibits glioma cell invasion by targeting E2F3. Mol Med Rep 2015; 11: 2838–44. 25515700 10.3892/mmr.2014.3101

[29] Chang X , Sun Y , Han S , Zhu W , Zhang H , Lian S . MiR‐203inhibits melanoma invasive and proliferative abilities by targeting the polycomb group gene BMI1. Biochem Biophys Res Commun 2015; 456: 361–6. 25475727 10.1016/j.bbrc.2014.11.087

[30] Zhou X , Xu G , Yin C , Jin W , Zhang G . Down‐regulation of miR‐203 induced by Helicobacter pylori infection promotes the proliferation and invasion of gastric cancer by targeting CASK. Oncotarget 2014; 5: 11631. 25373785 10.18632/oncotarget.2600 PMC4294334

[31] Zhou M , Chen J , Zhou L , Chen W , Ding G , Cao L . Pancreatic cancer derived exosomes regulate the expression of TLR4 in dendritic cells via miR‐203. Cell Immunol 2014; 292: 65–9. 25290620 10.1016/j.cellimm.2014.09.004

[32] Wang N , Liang H , Zhou Y *et al* miR‐203 suppresses the proliferation and migration and promotes the apoptosis of lung cancer cells by targeting SRC. PLOS One 2014; 9: e105570. 25140799 10.1371/journal.pone.0105570 PMC4139332

[33] Chen Z , Li D , Cheng Q *et al* MicroRNA‐203 inhibits the proliferation and invasion of U251 glioblastoma cells by directly targeting PLD2. Mol Med Rep 2014; 9: 503–8. 24270883 10.3892/mmr.2013.1814

[34] Ralfkiaer U , Hagedorn PH , Bangsgaard N *et al* Diagnostic microRNA profiling incutaneous T‐cell lymphoma (CTCL). Blood 2011; 118: 5891–900. 21865341 10.1182/blood-2011-06-358382 PMC3342856

[35] Wang S , Zhao X , Wang J *et al* microRNA‐203 isassociated with advanced tumor progression and poor prognosis in epithelial ovarian cancer. Med Oncol 2013; 30: 681. 23918241 10.1007/s12032-013-0681-x

[36] Iorio MV , Visone R , Di Leva G *et al* MicroRNA signatures in human ovarian cancer. Cancer Res 2007; 67: 8699–707. 17875710 10.1158/0008-5472.CAN-07-1936

[37] Li Z , Du L , Dong Z *et al* MiR‐203 suppresses ZNF217 upregulation in colorectal cancer and its oncogenicity. PLOS One 2015; 10: e0116170. 25621839 10.1371/journal.pone.0116170 PMC4306553

[38] Yantiss RK , Goodarzi M , Zhou XK *et al* Clinical, pathologic, and molecular features of early‐onset colorectal carcinoma. Am J Surg Pathol 2009; 33: 572–82. 19047896 10.1097/PAS.0b013e31818afd6b

[39] Taipaleenmäki H , Browne G , Akech J *et al* Targeting of Runx2 by miR‐135 and miR‐203 impairs progression of breast cancer and metastatic bone disease. Cancer Res 2015; 75: 1433–44. 25634212 10.1158/0008-5472.CAN-14-1026 PMC4383679

[40] Wang C , Zheng X , Shen C , Shi Y . MicroRNA‐203 suppresses cell proliferation and migration by targeting BIRC5 and LASP1 in human triple‐negative breast cancer cells. J Exp Clin Cancer Res 2012; 31: 58. 22713668 10.1186/1756-9966-31-58 PMC3585778

[41] Zhang Z , Zhang B , Li W *et al* Epigenetic silencing of miR‐203 upregulates SNAI2 and contributes to the invasiveness of malignant breast cancer cells. Genes Cancer 2011; 2: 782–91. 22393463 10.1177/1947601911429743 PMC3278899

[42] Saini S , Arora S , Majid S *et al* Curcumin modulates microRNA‐203‐mediated regulation of the Src‐Akt axis in bladder cancer. Cancer Prev Res (Phila) 2011; 4: 1698–709. 21836020 10.1158/1940-6207.CAPR-11-0267 PMC3940389

[43] Gottardo F , Liu CG , Ferracin M *et al* Micro‐RNA profiling in kidney and bladder cancers. Urol Oncol 2007; 25: 387–92. 17826655 10.1016/j.urolonc.2007.01.019

[44] Tang R , Zhong T , Dang Y , Zhang X , Li P , Chen G . Association between downex‐pression of MiR‐203 and poor prognosis in non‐small cell lung cancer patients. Clin Transl Oncol 2016; 18 (4): 360–8. 26307752 10.1007/s12094-015-1377-9

[45] Wan D , Shen S , Fu S *et al* miR‐203 suppresses the prolifera‐tion and metastasis of hepatocellular carcinoma by targeting onco‐gene ADAM9 and oncogenic long non‐ coding RNA HULC. Anticancer Agents Med Chem 2016; 16 (4): 414–23. 26179263 10.2174/1871520615666150716105955

[46] Yuan Y , Gong DJG , Liu XHL *et al* MiR‐203 inhibition of proliferation and invasion of Eca109 cells in esophageal squamous cell carcinoma. Journal of the second Mil Med Univ 2010; 31 (8): 818–20.

